# Association of the triglyceride-glucose index and its related parameters with frailty

**DOI:** 10.1186/s12944-024-02147-4

**Published:** 2024-05-21

**Authors:** Huangyi Yin, Liuqing Guo, Wei Zhu, Weishan Li, Yubo Zhou, Wenyun Wei, Min Liang

**Affiliations:** 1 The First Affiliated Hospital of Guangxi Medical University, Nanning, China; 2 Department of Geriatric Endocrinology and Metabolism, The First Affiliated Hospital of Guangxi Medical University, No. 6 Shuangyong Road, Nanning, Guangxi 530021 China

**Keywords:** Triglyceride-glucose, Triglyceride glucose-body mass index, Triglyceride glucose-wait circumference, Triglyceride glucose-waist to height ratio, Frailty

## Abstract

**Background:**

Frailty is a dynamic geriatric condition. Limited studies have examined the association of the triglyceride-glucose (TyG) index and its related indicators [TyG index, triglyceride glucose-waist to height ratio (TyG-WHtR), triglyceride glucose-waist circumference (TyG-WC), and triglyceride glucose-body mass index (TyG-BMI)] with frailty, and the potential links among them remain unclear. On the basis of data from the National Health and Nutrition Examination Survey (NHANES), this study investigated the potential relationships of the TyG index and its related indices with frailty.

**Methods:**

This research included 7,965 participants from NHANES 2003–2018. The relationship of the TyG index and its related indices with frailty was investigated with binary logistic regression analyses, restricted cubic spline (RCS), and receiver operating characteristic (ROC) curve. Potential influences were further investigated through stratified analyses and interaction tests.

**Results:**

The prevalence of frailty in the participants of this study was 25.59%, with a average frailty index of 0.16 (0.00). In the three regression analysis models, the continuous TyG index and its associated indices were positively associated with frailty. In addition, quartiles of TyG, TyG-WC, TyG-WHtR, and TyG-BMI were significantly associated with increased frailty prevalence in the fully adjusted models (TyG Q4 vs. Q1, OR = 1.58, 95% CI: 1.19, 2.09, *P* = 0.002; TyG-WC Q4 vs. Q1, OR = 2.40, 95% CI: 1.90, 3.04, *P* < 0.001; TyG-WHtR Q4 vs. Q1, OR = 2.26, 95% CI: 1.82, 2.81, *P* < 0.001; TyG- BMI Q4 vs. Q1, OR = 2.16, 95% CI: 1.76, 2.64, *P* < 0.001). According to RCS analysis, TyG, TyG-WC, TyG-WHtR, and TyG-BMI were linearly and positively associated with frailty. ROC curves revealed that TyG-WHtR (AUC: 0.654) had greater diagnostic value for frailty than TyG (AUC: 0.604), TyG-BMI (AUC: 0.621), and TyG-WC (AUC: 0.629). All of the stratified analyses and interaction tests showed similar results.

**Conclusions:**

Elevated TyG and its associaed indices are associated with an increased prevalence of frailty. Reasonable control of blood glucose and blood lipids, and avoidance of obesity, may aid in reducing the occurrence of frailty in middle-aged and older adults.

**Supplementary Information:**

The online version contains supplementary material available at 10.1186/s12944-024-02147-4.

## Introduction

Frailty, a dynamic syndrome characterized by impaired functioning of physiological reserves and decreased vulnerability to external stresses, often occurs in older people [1]. Frailty affects 7–24% of older people and is notably associated with both diminished muscle mass and the occurrence of chronic illness [2, 3]. Frailty not only increases the risks of falls, disability, and hospitalization in older people, but also increases all-cause mortality, thus posing a major threat to public health [4–6]. Fortunately, frailty is not irreversible and can be partially alleviated by the treatment of chronic diseases [7]. The prevention and management of frailty in older people in an aging society continue to warrant the utmost attention.

Insulin resistance (IR), characterized by diminished sensitivity or responsiveness to insulin, is implicated in the etiology of numerous disorders, including non-alcoholic fatty liver disease (NAFLD), diabetes mellitus (DM), cardiovascular disease (CVD), and cognitive impairment [8–10]. The classic hyperinsulinemic-euglycemic clamp can accurately diagnose IR, but is expensive and cumbersome to perform, and therefore is inappropriate for widespread clinical application [11]. Consequently, researchers developed the homeostasis model assessment of insulin resistance (HOMA-IR) for clinical studies; however, this model is susceptible to exogenous insulin therapy and is not suitable for patients with β-cell incompetence [11, 12]. In addition, the triglyceride-glucose (TyG) index is easier to obtain than the HOMA-IR. The TyG index, which is based on fasting triglycerides and fasting glucose, has gained widespread acceptance as a reliable substitute indicator of IR in recent years for predicting CVD, DM, hypertension, stroke, and mortality [13–16]. Numerous studies have suggested that the diagnostic value of TyG for disease and IR is superior to that of the traditional indicator HOMA-IR [17–19]. In addition, the TyG index has been reported to be significantly correlated with declines in muscle mass and strength in older people [20, 21].

Both frailty and IR are closely associated with increasing age [22]. Given that comorbidities such as CVD, cognitive dysfunction, and loss of muscle mass are all critical determinants of frailty, the relationship between surrogate indicators of IR and frailty has received attention from researchers. Previous studies have shown that HOMA-IR is positively associated with frailty, suggesting that IR may be involved in the pathologic process of frailty [23, 24]. However, limited studies have examined the relationship between the TyG index and frailty. Recently, a prospective cohort study from China has suggested that an elevated TyG index is a potential risk factor for frailty, particularly in populations with high body mass index (BMI) [25]. Nevertheless, it is unclear if this conclusion applies to Americans. Obesity is prevalent worldwide and is more likely to occur in older than younger people [22]. Obesity increases the risk of CVD and cancer, and has also been reported to be associated with sarcopenia [26–28]. Waist circumference (WC), BMI, and waist-to-height ratio (WHtR) are considered reliable anthropometric measures for assessing obesity. Compared with the TyG index alone, a combination of obesity metrics with TyG has been demonstrated to increase the diagnostic value and relevance to diseases such as CVD, NAFLD, and DM [29–31]. Obesity is also an important factor influencing frailty [32, 33]. However, there are no studies exploring the association between TyG-related metrics [triglyceride glucose-waist to height ratio (TyG-WHtR), triglyceride glucose-waist circumference (TyG-WC), and triglyceride glucose-body mass index (TyG-BMI)] and frailty. And it is uncertain whether the diagnostic value of these indicators for frailty is superior to that of the TyG index. Therefore, we hypothesized that TyG and its related indices are associated with frailty and that TyG-related indicators may be more beneficial than the TyG index in the diagnosis of frailty.

Consequently, this study was aimed at performing what is, to our knowledge, the first investigation of the relationships of various TyG-related parameters with frailty, and to provide valuable insights into the association between IR and frailty.

## Methods

### Study design

The 80,312 participants in this study were from the 2003–2018 National Health and Nutrition Examination Survey (NHANES), a national study designed to investigate the physical and nutritional health of noninstitutionalized American residents through a complex probability sampling design. NHANES participants provided informed consent, and the study received United States National Center for Health Statistics Research Ethics Board approval, in accordance with the Declaration of Helsinki.

**Fig. 1:**
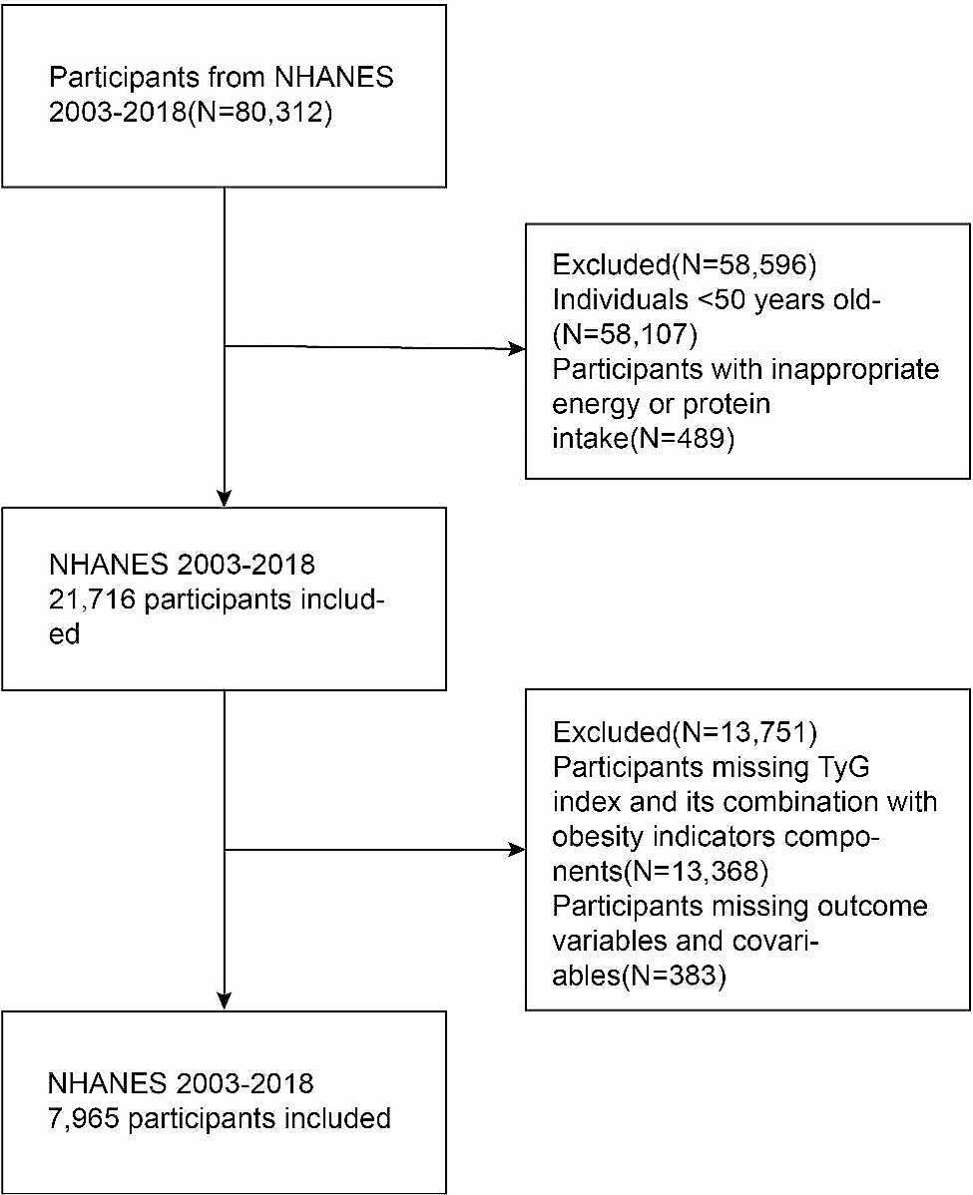
Flow chart of the participants exclusion process

Because frailty is prevalent in older people, this study was designed to investigate the middle-aged and older population above 50 years of age (*n* = 22,205). After exclusion of part of the population according to the following criteria, 7,965 individuals were available for inclusion in this study [none of these participants were missing the frailty index (FI)]. First, 489 individuals who consumed aberrant amounts of energy (< 500 kcal/day or ≥ 3500 kcal/day for women, or < 800 kcal/day or ≥ 4200 kcal/day for men) were excluded. Second, 13,368 participants with missing TyG and parameters of physical obesity measurements were excluded. Finally, 383 participants missing outcome variables and covariables were also excluded. Notably, because many participants had missing poverty income ratio (PIR), alcohol consumption, and physical activity (PA) (7.04%, 6.02%, and 26.25%, respectively) data, these participants were identified as the “unknown” group for the analyses (procedure in Fig. 1).

### Frailty index and frailty

Two methods are frequently used to assess frailty: FI and the frailty phenotype. Given the higher sensitivity of the FI in diagnosing frailty, for diagnosis, we used the FI developed by Rockwood et al., which is also the most common diagnostic method [34]. On the basis of previous NHANES studies, the FI involved 49 items regarding health deficits (covering eight dimensions of cognition, dependence, depression, comorbidity, anthropometry, laboratory tests, hospital utilization, and health status). FI is calculated as the number of actual defective items divided by the total number of defects, with a reference range of 0–1. A higher FI indicated greater frailty severity, and FI > 0.21 was considered to meet the diagnostic criteria for frailty [34–36]. Supplementary Table 1 shows the specific health deficit items and scores in detail.

### TyG and its related indicators

We collected data on height, weight, WC, fasting glucose, and fasting triglycerides for the calculation of TyG and its related indexes with the following formulas: WHtR = WC (cm)/height (cm); BMI = weight (kg)/height (m)^2^; TyG = ln (fasting triglyceride [mg/dl] * fasting glucose [mg/dl]/2); TyG-WC = TyG * WC (cm); TyG-BMI = TyG * BMI(kg/m^2^); and TyG-WHtR = TyG * WHtR. Measurements of fasting triglycerides and blood glucose required participants to remain fasting for more than 8 h.

### Covariables

This study included indicators that might potentially influence the associations of TyG and its related indicators with frailty, according to previous research. Demographic factors included sex, age, race, and education level. PIR was divided into < 1.3, 1.3–3.5, ≥ 3.5, and unknown. BMI was classified into three categories: <25 kg/m^2^, 25–30 kg/m^2^, and ≥ 30 kg/m^2^. Laboratory indicators included fasting total cholesterol (TC), high-density lipoprotein cholesterol (HDL-C) and low-density lipoprotein cholesterol (LDL-C). In addition, the mean energy intake from two 24-hour dietary recall questionnaires were included in the study.

Smoking was classified into three categories: never smoking, former smoking, and current smoking. Alcohol consumption status consisted of never drinking, previous drinking, current drinking (light, moderate, and heavy), and unknown. PA was represented by metabolic equivalents (MET), which were divided into ≤ 700 MET-min/week, 700-2400 MET-min/week, > 2400 MET-min/week, and unknown. Finally, data on use of hypoglycemic or hypolipidemic agents were collected from questionnaires. The Healthy Eating Index (HEI) developed by the US Department of Agriculture was used to assess dietary health; specifically, the HEI was assessed across 13 components, including 9 adequate and 4 appropriate components. This study was assessed using the mean of dietary components recorded from two 24-hour dietary recall interviews. Higher scores indicated healthier diets.

### Statistical analysis

To provide a comprehensive representation of the national sample of non-institutionalized residents, NHANES uses a complex multi-stage stratified probability survey design and recommends the use of appropriate weights for statistical analyses. Accordingly, all samples were weighted for this study. The ANOVA analysis and Kruskal-Wallis rank test was used for comparing continuous values, which are reported as mean (standard error). Categorical variables are represented by sample sizes (percentages), and the chi-square test was used for comparisons between groups. First, participants were grouped by the occurrence of frailty and quartiles of TyG and its related indices to compare baseline characteristics. Subsequently, three multifactorial regression analysis models were used to explore the association between TyG and its related indices and frailty. Multicollinearity of covariables in multifactorial regression analyses was assessed using the generalized variance-inflation factor (GVIF) function in the “car” package. Covariables with GVIF^(1/(2*df)) < 2 were considered to have less collinearity and were used in the final analyses. Model 1 was crude adjustment model; model 2 was adjusted for sex, race, and age; model 3 was adjusted for sex (male, female), age (continuous), race (non-Hispanic Black, non-Hispanic White, American Mexican, others), education level (below high school, high school graduate, college or above), PIR (< 1.3, 1.3–3.5, ≥ 3.5, and unknown), BMI (< 25 kg/m^2^, 25–30 kg/m^2^, and ≥ 30 kg/m^2^), PA (≤ 700 MET-min/week, 700-2400 MET-min/week, > 2,400 MET-min/week, and unknown), smoking status (never, former, and current), drinking status (never, previous, current (light, moderate, and heavy), and unknown), energy intake (continuous), HEI (continuous), TC (continuous), HDL-C (continuous), year (continuous), use of hypoglycemic agents (no, yes), and use of hypolipidemic agents (no, yes). To avoid covariance, TyG-WC, TyG-BMI, and TyG-WHtR was not adjusted for BMI in model 3. Restricted cubic spline (RCS) analysis was used to illustrate the relationships among frailty and TyG, TyG-WC, TyG-BMI, and TyG-WHtR after all covariables had been adjusted for. In addition, receiver operating characteristic (ROC) curves were used to evaluate the diagnostic value of TyG and its associated indices for frailty. Finally, stratified analyses and interaction tests were used to further explore the links of TyG, TyG-WC, TyG-WHtR, and TyG-BMI with frailty. This study performed a logarithmic transformation of the high values of TyG-WC and TyG-BMI during the multifactorial regression analysis, the stratified analysis and the examination of interactions. R4.3.2 software was used for all statistical analyses in this work, and *P* < 0.05 indicated statistical significance.

## Results

### Baseline features of individuals

A total of 7,965 samples were included in this study. The percentages of men and women was 46.03% and 53.97%, respectively, the average age was 63.09 (0.16) years, and the average FI was 0.16 (0.00). Furthermore, 25.59% of participants were diagnosed with frailty.

The participants’ baseline characteristics were compared in accordance with the quartile groupings of TyG, TyG-WC, TyG-WHtR, and TyG-BMI; the results are displayed in Table 1 and the supplementary files (Table S2, Table S3, Table S4). The participants with higher TyG index and its related indices had lower HDL-C, PA, PIR, and education levels; also, obese, with frailty people, and individuals taking hypoglycemic or hypolipidemic medications had higher TyG index and its related metrics.

**Table 1:** Comparison of weighted baseline characteristics of participants according to quartiles of the TyG index

Variables		Total *N* = 7965	TyG index				*P*-value
			Q1(≤ 8.33) *N* = 1991	Q2(8.33–8.72) *N* = 1992	Q3(8.72–9.14) *N* = 1991	Q4(≥ 9.14) *N* = 1991	
General Characteristics							
Gender (%)							0.005
Female	4075(53.97)		1068(58.02)	1026(54.86)	1006(52.71)	975(49.85)	
Male	3890(46.03)		923(41.98)	966(45.14)	985(47.29)	1016(50.15)	
Age (years)	63.09(0.16)		62.52(0.30)	63.34(0.28)	63.58(0.29)	62.96(0.25)	0.020
Race (%)							< 0.001
Non-Hispanic Black	1511(9.30)		611(14.53)	422(10.34)	264(6.41)	214(5.44)	
Non-Hispanic White	3926(76.10)		925(74.48)	967(75.87)	1024(77.23)	1010(76.97)	
American Mexican	1124(4.70)		155(2.58)	252(4.26)	321(5.36)	396(6.86)	
Others	1404(9.89)		300(8.41)	351(9.53)	382(11.00)	371(10.74)	
Education level (%)							< 0.001
Below high school	2213(16.61)		442(12.90)	503(14.96)	598(18.28)	670(20.70)	
High school graduate	1905(25.52)		435(21.48)	511(26.00)	446(25.45)	513(29.58)	
College or above	3847(57.88)		1114(65.62)	978(59.03)	947(56.27)	808(49.73)	
PIR (%)							< 0.001
< 1.3	1926(14.86)		409(12.25)	464(14.37)	493(15.75)	560(17.35)	
1.3–3.5	2936(34.18)		698(29.92)	754(34.39)	724(35.05)	760(37.79)	
≥ 3.5	2410(43.92)		707(49.96)	610(44.19)	596(42.56)	497(38.35)	
Unknown	693(7.04)		177(7.87)	164(7.05)	178(6.64)	174(6.51)	
Nutrition & life style							
HEI	55.81(0.26)		57.82(0.49)	55.71(0.46)	55.15(0.47)	54.36(0.39)	< 0.001
Energy intake (kcal/day)	1942.03 (10.71)		1950.57 (19.96)	1968.80 (23.32)	1906.64 (22.30)	1941.62 (19.38)	0.261
Drinking (%)							< 0.001
Never	1128(11.41)		269(10.93)	267(11.15)	299(11.09)	293(12.56)	
Former	1752(18.07)		379(15.60)	427(16.52)	433(18.62)	513(21.90)	
Mild	2899(41.90)		792(44.61)	758(43.34)	721(42.62)	628(36.61)	
Moderate	839(12.67)		238(14.95)	218(12.95)	189(10.36)	194(12.29)	
Heavy	796(9.92)		163(7.93)	185(9.91)	222(11.44)	226(10.58)	
Unknown	551(6.02)		150(5.98)	137(6.14)	127(5.88)	137(6.08)	
Smoking (%)							0.004
Never	3902(49.02)		1032(51.85)	989(51.05)	1000(49.34)	881(43.40)	
Former	2808(35.51)		677(34.11)	689(35.12)	675(33.62)	767(39.46)	
Now	1255(15.47)		282(14.04)	314(13.83)	316(17.04)	343(17.14)	
PA (MET-mins/week)							< 0.001
≤ 700	1818(23.86)		453(23.53)	461(23.57)	445(23.21)	459(25.21)	
700–2400	1851(25.29)		499(27.26)	459(25.29)	487(26.09)	406(22.24)	
> 2400	1783(24.60)		509(28.77)	483(26.25)	400(22.06)	391(20.91)	
Unknown	2513(26.25)		530(20.44)	589(24.89)	659(28.64)	735(31.64)	
Medical condition							
Lipid-lowering agents (%)							< 0.001
No	5036(63.31)		1377(71.03)	1312(66.30)	1223(60.54)	1124(54.49)	
Yes	2929(36.69)		614(28.97)	680(33.70)	768(39.46)	867(45.51)	
Hypoglycemic agents (%)							< 0.001
No	6452(84.68)		1821(94.27)	1747(91.47)	1611(84.61)	1273(66.93)	
Yes	1513(15.32)		170(5.73)	245(8.53)	380(15.39)	718(33.07)	
Frailty (%)							< 0.001
No	5559(74.41)		1517(81.94)	1468(77.86)	1382(73.28)	1192(63.56)	
Yes	2406(25.59)		474(18.06)	524(22.14)	609(26.72)	799(36.44)	
FI	0.16(0.00)		0.14(0.00)	0.15(0.00)	0.17(0.00)	0.19(0.00)	< 0.001
Physical Exam & Laboratory data							
BMI (kg/m^2^)							< 0.001
< 25	2027(26.41)		797(43.09)	563(28.52)	392(19.89)	275(12.47)	
25–30	2833(35.05)		663(33.40)	770(38.50)	724(35.24)	676(33.06)	
≥ 30	3105(38.54)		531(23.52)	659(32.98)	875(44.87)	1040(54.47)	
TC (mmol/l)	5.16(0.02)		4.90(0.03)	5.12(0.03)	5.20(0.04)	5.43(0.04)	< 0.001
HDL-C (mmol/l)	1.45(0.01)		1.75(0.02)	1.52(0.01)	1.35(0.01)	1.16(0.01)	< 0.001
LDL-C (mmol/l)	3.02(0.02)		2.83(0.03)	3.09(0.03)	3.13(0.03)	3.06(0.03)	< 0.001

Continuous variables were expressed as mean (standard error) and categorical variables were expressed as frequencies (percentages)

TyG: triglyceride-glucose; PIR: poverty income ratio; HEI: Healthy Eating Index; PA: physical activity; MET: metabolic equivalents; FI: frailty index; BMI: body mass index; TC: total cholesterol; HDL-C: high-density lipoprotein cholesterol; LDL-C: low-density lipoprotein cholesterol

When grouped by frailty, the results showed that frail participants had higher age, TyG, TyG-BMI, TyG-WC, and TyG-WHR, while education level, PIR, PA, HEI, TC, HDL-C, LDL-C, and energy intake were typically lower compared to non-frail participants. In addition, the frail participants were more likely to be female, never drinkers, current smokers, obese individuals, individuals taking hypoglycemic or hypolipidemic agents (Table 2).

**Table 2:** Comparison of weighted baseline characteristics of participants according to frailty

Variables	Total (*N* = 7965)	Non-frailty (*N* = 5559)	Frailty (*N* = 2406)	*P*-value
General Characteristics				
Sex (%)				< 0.001
Female	4075(53.97)	2681(51.31)	1394(61.72)	
Male	3890(46.03)	2878(48.69)	1012(38.28)	
Age (years)	63.09(0.16)	62.44(0.17)	65.01(0.28)	< 0.001
Race (%)				< 0.001
Non-Hispanic Black	1511(9.30)	957(7.81)	554(13.65)	
Non-Hispanic White	3926(76.10)	2773(77.94)	1153(70.76)	
American Mexican	1124(4.70)	791(4.53)	333(5.22)	
Others	1404(9.89)	1038(9.72)	366(10.37)	
Education level (%)				< 0.001
Below high school	2213(16.61)	1316(13.02)	897(27.03)	
High school graduate	1905(25.52)	1295(24.21)	610(29.33)	
College or above	3847(57.88)	2948(62.78)	899(43.64)	
PIR (%)				< 0.001
< 1.3	1926(14.86)	1064(10.33)	862(28.02)	
1.3–3.5	2936(34.18)	2020(32.00)	916(40.51)	
≥ 3.5	2410(43.92)	1995(50.54)	415(24.69)	
Unknown	693(7.04)	480(7.12)	213(6.77)	
Nutrition & life style				
HEI	55.81(0.26)	56.72(0.31)	53.13(0.38)	< 0.001
Energy intake (kcal/day)	1942.03(10.71)	1984.95(12.65)	1817.26(20.38)	< 0.001
Drinking (%)				< 0.001
Never	1128(11.41)	752(10.67)	376(13.55)	
Former	1752(18.07)	1068(15.12)	684(26.66)	
Mild	2899(41.90)	2224(45.35)	675(31.86)	
Moderate	839(12.67)	627(13.49)	212(10.29)	
Heavy	796(9.92)	561(10.19)	235(9.15)	
Unknown	551(6.02)	327(5.17)	224(8.48)	
Smoking (%)				< 0.001
Never	3902(49.02)	2850(51.59)	1052(41.54)	
Former	2808(35.51)	1942(35.44)	866(35.70)	
Now	1255(15.47)	767(12.97)	488(22.76)	
PA (MET-mins/week)				< 0.001
≤ 700	1818(23.86)	1286(24.28)	532(22.64)	
700–2400	1851(25.29)	1440(27.58)	411(18.62)	
> 2400	1783(24.60)	1424(27.71)	359(15.56)	
Unknown	2513(26.25)	1409(20.43)	1104(43.18)	
Medical condition				
Lipid-lowering agents (%)				< 0.001
No	5036(63.31)	3861(68.62)	1175(47.88)	
Yes	2929(36.69)	1698(31.38)	1231(52.12)	
Hypoglycemic agents (%)				< 0.001
No	6452(84.68)	4890(90.01)	1562(69.18)	
Yes	1513(15.32)	669(9.99)	844(30.82)	
FI	0.16(0.00)	0.12(0.00)	0.30(0.00)	< 0.001
TyG	8.74(0.01)	8.68(0.01)	8.91(0.02)	< 0.001
TyG-WC	894.25(3.54)	874.09(3.36)	952.86(6.05)	< 0.001
TyG-WHR	5.35(0.02)	5.20(0.02)	5.77(0.04)	< 0.001
TyG-BMI	256.45(1.36)	249.14(1.30)	277.71(2.32)	< 0.001
Physical Exam & Laboratory data				
BMI (%)				< 0.001
< 25 kg/m^2^	2027(26.41)	1579(28.86)	448(19.29)	
25–30 kg/m^2^	2833(35.05)	2096(36.63)	737(30.46)	
≥ 30 kg/m^2^	3105(38.54)	1884(34.51)	1221(50.25)	
TC (mmol/l)	5.16(0.02)	5.22(0.02)	4.98(0.04)	< 0.001
HDL-C (mmol/l)	1.45(0.01)	1.48(0.01)	1.38(0.02)	< 0.001
LDL-C (mmol/l)	3.02(0.02)	3.09(0.02)	2.82(0.03)	0.001

Continuous variables were expressed as mean (standard error) and categorical variables were expressed as frequencies (percentages)

PIR: poverty income ratio; HEI: Healthy Eating Index; PA : physical activity; MET: metabolic equivalents; FI: frailty index; TyG: triglyceride-glucose; TyG-WC: triglyceride glucose-waist circumference; TyG-WHtR: triglyceride glucose-waist to height ratio; TyG-BMI: triglyceride glucose-body mass index; BMI: body mass index; TC: total cholesterol; HDL-C: high-density lipoprotein cholesterol; LDL-C: low-density lipoprotein cholesterol

### Association of TyG and its related metrics with frailty

**Fig. 2:**
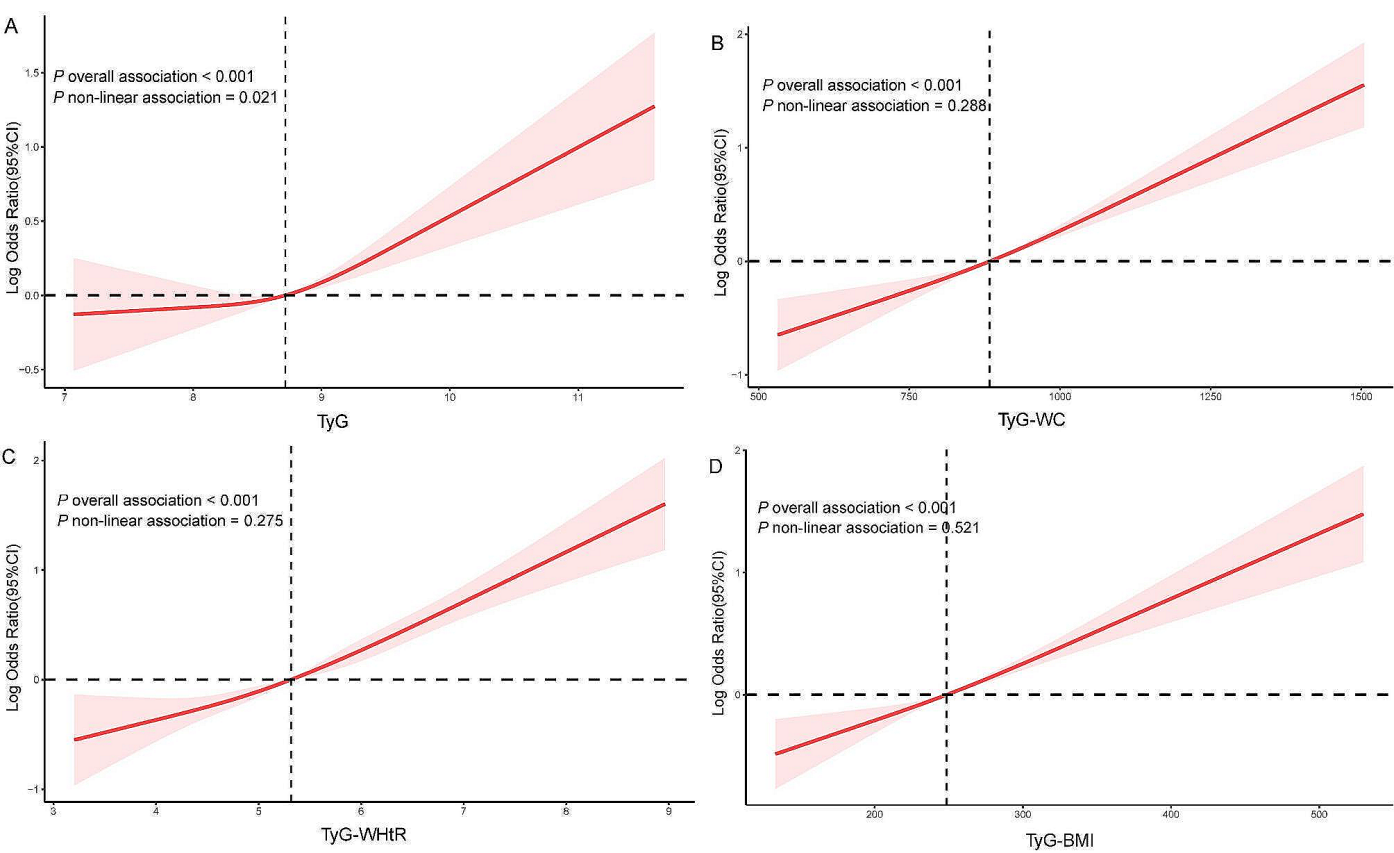
Restricted cubic spline fitting for the association between TyG and its associated indices and frailty in model 3

**Fig. 3:**
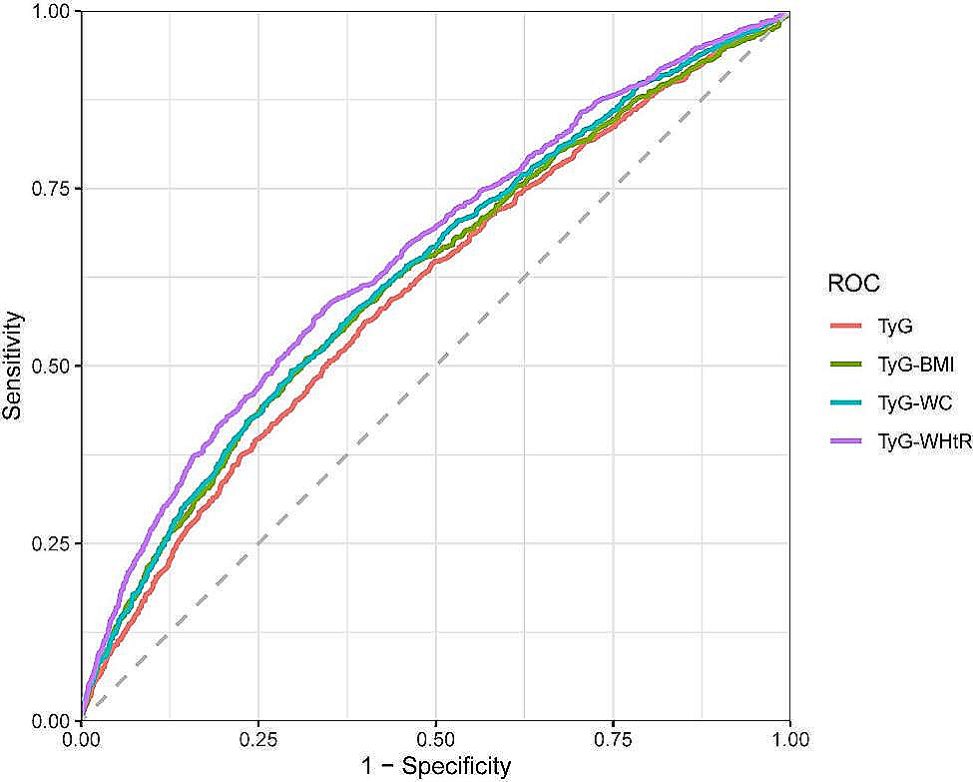
ROC curves of the TyG index and its indices in relation to frailty

LDL-C was not included in the adjustment model owing to the presence of collinearity (GVIF^(1/(2*df)) > 2). As is shown in the Table 3, higher TyG, TyG-WC, TyG-WHtR, and TyG-BMI were significantly associated with a higher prevalence of frailty in all three multifactorial regression models. In the fully adjusted model, participants in the fourth quartile of TyG, TyG-WC, TyG-WHtR, and TyG-BMI were significantly more likely to develop frailty compared with participants in the first quartile (TyG Q4 vs. Q1, OR = 1.58, 95% CI: 1.19, 2.09, *P* = 0.002; TyG-WC Q4 vs. Q1, OR = 2.40, 95% CI: 1.90, 3.04, *P* < 0.001; TyG-WHtR Q4 vs. Q1, OR = 2.26, 95% CI: 1.82, 2.81, *P* < 0.001; TyG- BMI Q4 vs. Q1, OR = 2.16, 95% CI: 1.76, 2.64, *P* < 0.001). After adjustment for all covariables, the RCS indicated linear positive associations of TyG, TyG-WC, TyG-WHtR, and TyG-BMI with frailty (Fig. 2). Furthermore, ROC curves were used to evaluate the diagnostic value of TyG and its relevant metrics for frailty. TyG-WHtR had the highest diagnostic value for frailty, and was followed by TyG-WC and TyG-BMI, whereas the TyG index had the lowest diagnostic value (AUC = 0.654, 0.629, 0.621, and 0.604, respectively) (Fig. 3).

**Table 3:** Association between TyG and related indicators with frailty

Exposures	Model1[OR (95% CI) *P*-value]	Model2[OR (95% CI) *P*-value]	Model3[OR (95% CI) *P*-value]
TyG (continuous)	1.78(1.62,1.97) < 0.001	1.97(1.77,2.19) < 0.001	1.36(1.16,1.59) < 0.001
TyG (quartiles)			
Q1	ref	ref	ref
Q2	1.29(1.05,1.58) 0.014	1.33(1.08,1.64) 0.007	1.12(0.90,1.40) 0.293
Q3	1.65(1.35,2.03) < 0.001	1.79(1.44,2.22) < 0.001	1.21(0.96,1.53) 0.101
Q4	2.60(2.19,3.08) < 0.001	2.98(2.48,3.59) < 0.001	1.58(1.19,2.09) 0.002
*P* for trend	< 0.001	< 0.001	0.002
Log TyG-WC (continuous)	12.92 (8.80,18.96) < 0.001	21.49(14.12,32.71) < 0.001	7.79(4.75,12.78) < 0.001
TyG-WC (quartiles)			
Q1	ref	ref	ref
Q2	1.33(1.05,1.68) 0.018	1.43(1.13,1.82) 0.003	1.26(0.99,1.62) 0.064
Q3	1.76(1.43,2.15) < 0.001	2.00(1.61,2.49) < 0.001	1.49(1.18,1.89) < 0.001
Q4	3.16(2.62,3.80) < 0.001	3.93(3.23,4.77) < 0.001	2.40(1.90,3.04) < 0.001
*P* for trend	< 0.001	< 0.001	< 0.001
TyG-WHtR (continuous)	1.78(1.66,1.91) < 0.001	1.79(1.67,1.92) < 0.001	1.46(1.34,1.60) < 0.001
TyG-WHtR (quartiles)			
Q1	ref	ref	ref
Q2	1.42(1.19,1.70) < 0.001	1.40(1.17,1.68) < 0.001	1.21(0.99,1.48) 0.068
Q3	1.77(1.44,2.17) < 0.001	1.70(1.39,2.09) < 0.001	1.28(1.02,1.59) 0.032
Q4	3.98(3.35,4.74) < 0.001	4.00(3.36,4.75) < 0.001	2.26(1.82,2.81) < 0.001
*P* for trend	< 0.001	< 0.001	< 0.001
Log TyG-BMI (continuous)	6.23(4.58,8.47) < 0.001	7.33(5.31,10.12) < 0.001	3.78(2.70,5.29) < 0.001
TyG-BMI (quartiles)			
Q1	ref	ref	ref
Q2	1.17(0.97,1.42) 0.101	1.21(0.99,1.47) 0.056	1.16(0.93,1.43) 0.187
Q3	1.70(1.38,2.10) < 0.001	1.79(1.44,2.22) < 0.001	1.46(1.14,1.87) 0.003
Q4	2.91(2.42,3.50) < 0.001	3.22(2.66,3.90) < 0.001	2.16(1.76,2.64) < 0.001
*P* for trend	< 0.001	< 0.001	< 0.001

Model 1 did not adjust for any covariables. Model 2 adjusted for gender, age and race. model 3 was adjusted for sex, age, race, education level, PIR, BMI, PA, smoking status, drinking status, energy intake, HEI, TC, HDL-C, year, use of hypoglycemic, and use of hypolipidemic agents (TyG-WC,TyG-BMI,TyG-WHtR was not adjusted for BMI)

TyG: triglyceride-glucose; TyG-BMI: triglyceride glucose-body mass index; TyG-WC: triglyceride glucose-waist circumference; TyG-WHtR: triglyceride glucose-waist to height ratio

### Stratified analyses

**Fig. 4:**
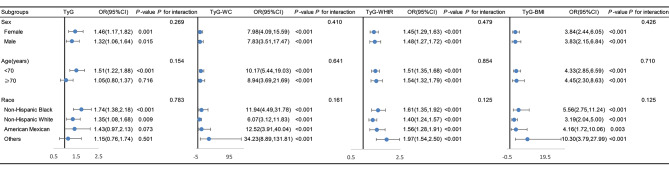
Subgroup analysis and interaction test of the association between TyG and its associated indices and frailty

Stratified analyses and interaction tests were used to further explore the association of TyG and its related indexes with frailty (Fig. 4). After stratification by sex, age, and race, no significant interactions were observed in all strata.

## Discussion

This large cross-sectional study provides the first insights into the links of TyG and its related indices with frailty. In this study, 25.59% of participants were diagnosed with frailty. After adjustment for all confounders, higher TyG, TyG-WC, TyG-WHtR, and TyG-BMI were all associated with a higher prevalence of frailty; this positive relationship was further validated in RSC. Further subgroup analyses suggested that this positive relationship remained stable across all subgroups. ROC curves showed that TyG-WHtR and TyG-WC had the highest diagnostic value for frailty.

The current findings indicated that the TyG index was positively associated with the prevalence of frailty. IR implies diminished sensitivity of the body to insulin and elevated plasma glucose levels, as well as a diminished ability of the liver to process glucose, thus resulting in the conversion of excess glucose into triglycerides. Therefore, the TyG index is considered a credible indicator of pancreatic β-cell function and is not influenced by the state of glucose metabolism [37]. The TyG index has been shown to have favorable specificity and sensitivity in the diagnosis and prediction of many diseases, particularly CVD [38–40]. In agreement with our findings, a recent two-way cohort study in China has indicated that an elevated TyG index and steady trajectory of the TyG index are beneficial regarding the development of frailty [25]. To our knowledge, that study has provided the only exploration of the relationship between the TyG index and frailty, although it is limited to Chinese individuals. Despite the scarcity of reports on the relationship between the TyG index and frailty, considerably strong evidence supports a positive association between them. The loss of muscle mass or strength and the development of chronic diseases, such as CVD and cognitive impairment, are the main contributors to frailty in older people. The TyG index has been reported to be positively related to sarcopenia, in populations with hypertension or without DM, as well as in adolescents or older people [21, 41–43]. Moreover, an elevated TyG index has been suggested to lead to elevated risk of CVD and mortality in cross-sectional or prospective studies [44–46]. Furthermore, cognitive impairment, an important factor in the evaluation of FI, has also been demonstrated to be positively associated with TyG [47–50]. A meta-analysis has revealed that an elevated TyG index is related to the frequency of cognitive impairment and dementia [47]. Importantly, prospective cohort studies have shown that HOMA-IR is positively associated with the risk of frailty in non-diabetic patients, suggesting that IR may be an important risk factor for frailty [23].

The potential mechanism underlying the relationship between the TyG index and frailty is unclear but may involve the following aspects: (1) Physiologically, insulin regulates glucose metabolism primarily in skeletal muscle [51]. Increasing age is often accompanied by declining hormone levels, decreased PA, and diminished skeletal muscle mass and ability to process glucose, thereby leading to a decrease in sensitivity to insulin [52]. Simultaneously, IR leads to increased breakdown of muscle proteins, thus creating a vicious cycle that ultimately induces frailty [53]. (2) With increasing age, the ability to process senescent organelles and misfolded proteins declines [54], and the levels of chronic inflammation and oxidative stress increase [55, 56], thus leading to IR and senescent disorders such as sarcopenia, CVD, cognitive disorders, and cancer [57–60]. Ultimately, the ability to resist external attacks is weakened.

This study performed the first investigation of the associations of TyG-WC, TyG-WHtR, and TyG-BMI with frailty. Interestingly, the diagnostic value of TyG-WC, TyG-WHtR, and TyG-BMI with frailty were stronger than those of the TyG index. BMI, WC, and WHtR are the most commonly used body measurements to assess obesity. The novel index constructed by TyG in combination with body measurements, compared with TyG alone, has shown better diagnostic value for multiple diseases [61, 62]. In addition, TyG in combination with body measurements is more accurate in evaluating IR than TyG alone [63–66]. Although studies exploring the connection between TyG-associated indices and frailty are limited, a relationship between them appears reasonable. The high prevalence of obesity threatens health among older people. Obesity induces skeletal muscle immune cell infiltration and the secretion of inflammatory factors from visceral adipose tissue, thereby inducing skeletal muscle inflammation, inhibiting myocyte metabolism, and leading to IR and ultimately multiple adverse outcomes [67]. A large cohort study has emphasized the importance of overweight or obesity in the relationship between the TyG index and frailty [25]. A Japanese study has suggested that obesity is beneficial regarding frailty in middle-aged and older adults, and appropriate weight loss can effectively decrease the prevalence of frailty in overweight and obese patients with diabetes [32].In addition, another cohort study has emphasized that decreasing abdominal obesity is a crucial measure for preventing frailty [33]. Furthermore, obesity is involved in the pathological mechanisms of diseases such as sarcopenia, CVD, and cognitive disorders, which are considered important components of frailty.

Notably, in our study, TyG-WHtR and TyG-WC had the highest diagnostic value for frailty. Body measurements representing abdominal obesity (WC and WHtR) have been demonstrated to be superior to BMI in the diagnosis of frailty [68–70]. These findings may be associated with the age-associated accumulation of abdominal fat causing increased levels of oxidative stress and chronic inflammation, both of which are detrimental to muscle anabolism and motor neuron repair, thus further aggravating dynapenia [71–74]. The best association between TyG-WC and CVD has also been reported, and TyG-WHtR has been found to be the best predictor of CVD mortality [44]. Therefore, the body adiposity distribution may be potentially related to frailty and its components.

### Strengths and limitations

This study has several strengths. First, this large study used the NHANES database, which is representative of the health conditions of the entire American population. Additionally, subgroup analyses and ROC curves were applied to further assess associations and diagnostic value of TyG and its associated indices. Importantly, this study is not only the first to explore the potential association between the TyG index and the prevalence of frailty in Americans, but also the first to investigate the relationship of TyG-associated indices with frailty. However, this study has several limitations. First, causality between TyG and its obesity-associated indices and frailty could not be determined, because this was a cross-sectional study. Second, this study was retrospective, and the possibility of remnant confounders cannot be completely excluded. Finally, this study population was limited to Americans, and the conclusions might not apply to other populations.

## Conclusions

This study suggests that elevated TyG index and its associated indices are associated with an increased prevalence of frailty. However, TyG-associated indicators, particularly TyG-WHtR and TyG-WC, performed best in the diagnosis of frailty. Consequently, we advocate for active glycemic and lipid control in middle-aged and older adults, as well as paying close attention to physical obesity, particularly abdominal obesity, may contribute to reducing the prevalence of frailty.

### Electronic supplementary material

Below is the link to the electronic supplementary material.

Supplementary Material 1

## Data Availability

No datasets were generated or analysed during the current study.

## Abbreviations

TyG: Triglyceride-glucose
NHANES: The National Health and Nutrition Examination Survey
RCS: Restricted cubic spline
ROC: Receiver operating characteristic
TyG-WC: Triglyceride glucose-waist circumference
TyG-WHtR: Triglyceride glucose-waist to height ratio
TyG-BMI: Triglyceride glucose-body mass index
IR: Insulin resistance
CVD: Cardiovascular disease
DM: Diabetes mellitus
NAFLD: Non-alcoholic fatty liver disease
HOMA-IR: Homeostasis model assessment of insulin resistance
BMI: Body mass index
WC: Waist circumference
WHtR: Waist-to-height ratio
FI: Frailty index
PIR: Poverty income ratio
TC: Total cholesterol
HDL-C: High-density lipoprotein cholesterol
LDL-C: Low-density lipoprotein cholesterol
PA: Physical activity
MET: Metabolic equivalents
HEI: Healthy Eating Index
GVIF: Generalized variance-inflation factor
