# Supplementary material for: Association of the triglyceride-glucose index and its related parameters with frailty
Source: Lipids Health Dis. 2024 May 21;23:150. doi: 10.1186/s12944-024-02147-4 (PMC11107008; doi:10.1186/s12944-024-02147-4)
Supplement: Supplementary file 1 — Supplementary Material 1 [file 12944_2024_2147_MOESM1_ESM.docx]

Supplementary Table 1. Variables and scorings in the 49-Item Frailty Index

| **Items** | | **scores** |
| --- | --- | --- |
| **Cognition** | |  |
| 1.experience confusion/memory problems | | yes=1, no=0 |
| **Dependence** | |  |
| 2.managing money difficulty | | no difficulty=0, Some difficulty=0.33, much difficulty=0.66, unable to do=1 |
| 3.walking for a quarter mile difficulty | | no difficulty=0, Some difficulty=0.33, much difficulty=0.66, unable to do=1 |
| 4.walking up ten steps difficulty | | no difficulty=0, Some difficulty=0.33, much difficulty=0.66, unable to do=1 |
| 5.stooping, crouching, kneeling difficulty | | no difficulty=0, Some difficulty=0.33, much difficulty=0.66, unable to do=1 |
| 6.lifting or carrying difficulty | | no difficulty=0, Some difficulty=0.33, much difficulty=0.66, unable to do=1 |
| 7.house chore difficulty | | no difficulty=0, Some difficulty=0.33, much difficulty=0.66, unable to do=1 |
| 8.preparing meals difficulty | | no difficulty=0, Some difficulty=0.33, much difficulty=0.66, unable to do=1 |
| 9.standing up from armless chair difficulty | | no difficulty=0, Some difficulty=0.33, much difficulty=0.66, unable to do=1 |
| 10.getting in and out of bed difficulty | | no difficulty=0, Some difficulty=0.33, much difficulty=0.66, unable to do=1 |
| 11.using fork, knife, drinking from cup difficulty | | no difficulty=0, Some difficulty=0.33, much difficulty=0.66, unable to do=1 |
| 12.dressing yourself difficulty | | no difficulty=0, Some difficulty=0.33, much difficulty=0.66, unable to do=1 |
| 13.standing for long periods difficulty | | no difficulty=0, Some difficulty=0.33, much difficulty=0.66, unable to do=1 |
| 14.grasp/holding small objects difficulty | | no difficulty=0, Some difficulty=0.33, much difficulty=0.66, unable to do=1 |
| 15.attending social event difficulty | | no difficulty=0, Some difficulty=0.33, much difficulty=0.66, unable to do=1 |
| 16.leisure activity at home difficulty | | no difficulty=0, Some difficulty=0.33, much difficulty=0.66, unable to do=1 |
| 17.push or pull large objects difficulty | | no difficulty=0, Some difficulty=0.33, much difficulty=0.66, unable to do=1 |
| **Depressive Conditions** | |  |
| 18.have little interest in doing things | nearly every day = 1, more than half the days = 0.66, several days = 0.33, no =0 | |
| 19.feeling down, depressed, or hopeless | nearly every day = 1, more than half the days = 0.66, several days = 0.33, no =0 | |
| 20.trouble sleeping or sleeping too much | nearly every day = 1, more than half the days = 0.66, several days = 0.33, no =0 | |
| 21.feeling tired or having little energy | nearly every day = 1, more than half the days = 0.66, several days = 0.33, no =0 | |
| 22.poor appetite or overeating | nearly every day = 1, more than half the days = 0.66, several days = 0.33, no =0 | |
| 23.feeling bad about yourself | nearly every day = 1, more than half the days = 0.66, several days = 0.33, no =0 | |
| 24.trouble concentrating on things | nearly every day = 1, more than half the days = 0.66, several days = 0.33, no =0 | |
| **Comorbidities** | |  |
| 25.doctor ever said you had arthritis | | yes = 1, no = 0 |
| 26.ever told you had thyroid problem | | yes = 1, no = 0 |
| 27.ever told you had chronic bronchitis | | yes = 1, no = 0 |
| 28.ever told you had cancer or malignancy | | yes = 1, no = 0 |
| 29.ever told had congestive heart failure | | yes = 1, no = 0 |
| 30.ever told you had coronary heart disease | | yes = 1, no = 0 |
| 31.ever told you had angina/angina pectoris | | yes = 1, no = 0 |
| 32.ever told you had heart attack | | yes = 1, no = 0 |
| 33.ever told you had a stroke | | yes = 1, no = 0 |
| 34.ever told you had high blood pressure | | yes = 1, no = 0 |
| 35.doctor told you have diabetes | | yes = 1, borderline=0.5, no =0 |
| 36.ever told you had weak/failing kidneys | | yes = 1, no =0 |
| 37.urine leakage bother you? | | greatly = 1, very much =0.75, somewhat= 0.5, only a little = 0.25, no=0 |
| **Hospital and Care** | |  |
| 38.general health condition | | excellent, very good, good = 0, fair, poor = 1 |
| 39.health now compared with 1 year ago | | worse =1, better = 0 |
| 40.overnight hospital patient in last year | | yes =1, no =0 |
| 41.times receive healthcare over past year | | no=0, 1-4=0.5, ≥5 =1 |
| 42.number of prescription medicines taken | | no =0, 1-4=0.5, ≥5 =1 |
| **Physical Anthropometry** | |  |
| 43.body mass index (kg/m^2^) | | <18.5, ≥30=1  ≥25, <30=0.5  ≥18.5,＜25=0 |
| **Laboratory values** | |  |
| 44.glycohemoglobin (%) | | 0%-5.7%=0, >5.7%=1 |
| 45.red blood cell count (million cells/ul) | | M: ≥4.7, <6.1=0, Other=1  F: ≥4.2,＜5.4=0, Other =1 |
| 46.hemoglobin (g/dl) | | M: ≥13.5, <18 =0, Other =1  F: ≥12, <16 =0, Other=1 |
| 47.red cell distribution width (%) | | ≥11.6, <14.6=0, Other=1 |
| 48.lymphocyte percent (%) | | ≥20, <40=0, Other=1 |
| 49.segmented neutrophils percent (%) | | ≥40, <80=0, Other=1 |

Supplementary Table 2. Comparison of weighted baseline characteristics of participants according to quartiles of TyG-WC

| Variables | Total  N=7965 | TyG-WC index | | | | | *P*-value |
| --- | --- | --- | --- | --- | --- | --- | --- |
|  |  | Q1(≤784.43)  N=1991 | Q2(784.43-883.42) N=1992 | | Q3(883.42-995.42) N=1990 | Q4(≥995.42)  N=1992 |  |
| General Characteristics | | | | | | | |
| Gender (%) |  |  |  |  | |  | <0.001 |
| Female | 4075(53.97) | 1253(69.30) | 992(52.29) | 944(48.76) | | 886(44.30) |  |
| Male | 3890(46.03) | 738(30.70) | 1000(47.71) | 1046(51.24) | | 1106(55.70) |  |
| Age (years) | 63.09(0.16) | 62.69(0.31) | 63.442(0.29) | 63.401(0.27) | | 62.891(0.25) | 0.093 |
| Race (%) |  |  |  |  | |  | <0.001 |
| Non-Hispanic Black | 1511(9.30) | 412(9.71) | 392(10.22) | 367(9.24) | | 340(8.07) |  |
| Non-Hispanic White | 3926(76.10) | 958(74.54) | 931(73.97) | 957(77.09) | | 1080(78.85) |  |
| American Mexican | 1124(4.70) | 175(2.84) | 276(4.54) | 357(6.17) | | 316(5.44) |  |
| Others | 1404(9.89) | 446(12.91) | 393(11.27) | 309(7.50) | | 256(7.63) |  |
| Education level (%) |  |  |  |  | |  | <0.001 |
| Below high school | 2213(16.61) | 457(13.57) | 532(15.80) | 631(20.04) | | 593(17.32) |  |
| High school graduate | 1905(25.52) | 476(24.21) | 471(24.72) | 473(25.40) | | 485(27.77) |  |
| College or above | 3847(57.88) | 1058(62.21) | 989(59.48) | 886(54.57) | | 914(54.91) |  |
| PIR (%) |  |  |  |  | |  | <0.001 |
| <1.3 | 1926(14.86) | 424(12.62) | 461(15.16) | 498(14.45) | | 543(17.33) |  |
| 1.3-3.5 | 2936(34.18) | 684(29.86) | 738(32.95) | 747(36.49) | | 767(37.73) |  |
| ≥3.5 | 2410(43.92) | 693(49.79) | 625(44.76) | 572(41.63) | | 520(39.10) |  |
| Unknown | 693(7.04) | 190(7.73) | 168(7.13) | 173(7.44) | | 162(5.83) |  |
| Nutrition & life style | | | | | | | |
| HEI | 55.81(0.26) | 58.30(0.52) | 56.78(0.39) | 55.10(0.42) | | 52.92(0.35) | <0.001 |
| Energy intake (kcal/day) | 1942.03  (10.71) | 1868.39  (17.45) | 1913.71  (20.83) | 1961.06  (21.15) | | 2028.82  (20.33) | <0.001 |
| Drinking (%) |  |  |  |  | |  | <0.001 |
| Never | 1128(11.41) | 325(12.69) | 276(10.68) | 281(11.76) | | 246(10.41) |  |
| Former | 1752(18.07) | 361(14.51) | 403(16.34) | 466(18.85) | | 522(22.76) |  |
| Mild | 2899(41.90) | 780(44.25) | 764(44.34) | 693(41.14) | | 662(37.81) |  |
| Moderate | 839(12.67) | 228(14.06) | 221(13.19) | 199(11.73) | | 191(11.61) |  |
| Heavy | 796(9.92) | 153(8.73) | 182(9.22) | 218(10.47) | | 243(11.34) |  |
| Unknown | 551(6.02) | 144(5.76) | 146(6.23) | 133(6.04) | | 128(6.06) |  |
| Smoking (%) |  |  |  |  | |  | <0.001 |
| Never | 3902(49.02) | 1078(53.61) | 1044(52.87) | 953(46.37) | | 827(43.02) |  |
| Former | 2808(35.51) | 563(29.03) | 646(31.93) | 748(39.92) | | 851(41.57) |  |
| Now | 1255(15.47) | 350(17.35) | 302(15.20) | 289(13.72) | | 314(15.41) |  |
| PA (MET-mins/week) |  |  |  |  | |  | <0.001 |
| ≤700 | 1818(23.86) | 484(23.98) | 453(23.47) | 443(24.07) | | 438(23.89) |  |
| 700-2400 | 1851(25.29) | 522(28.45) | 465(24.89) | 449(24.01) | | 415(23.52) |  |
| >2400 | 1783(24.60) | 473(27.26) | 469(26.28) | 455(25.16) | | 386(19.67) |  |
| Unknown | 2513(26.25) | 512(20.30) | 605(25.35) | 643(26.76) | | 753(32.92) |  |
| Medical condition | | | | | | | |
| Lipid-lowering agents (%) |  |  |  |  | |  | <0.001 |
| No | 5036(63.31) | 1468(73.95) | 1281(66.45) | 1190(58.73) | | 1097(53.43) |  |
| Yes | 2929(36.69) | 523(26.05) | 711(33.55) | 800(41.27) | | 895(46.57) |  |
| Hypoglycemic agents (%) |  |  |  |  | |  | <0.001 |
| No | 6452(84.68) | 1866(95.89) | 1735(90.61) | 1557(83.33) | | 1294(68.47) |  |
| Yes | 1513(15.32) | 125(4.11) | 257(9.39) | 433(16.67) | | 698(31.53) |  |
| Frailty (%) |  |  |  |  | |  | <0.001 |
| No | 5559(74.41) | 1601(83.27) | 1503(78.92) | 1360(73.93) | | 1095(61.19) |  |
| Yes | 2406(25.59) | 390(16.73) | 489(21.08) | 630(26.07) | | 897(38.81) |  |
| FI | 0.16(0.00) | 0.14(0.00) | 0.15(0.00) | 0.17(0.00) | | 0.20(0.00) | <0.001 |
| Physical Exam & Laboratory data | | | | | | | |
| BMI (kg/m^2^) |  |  |  |  | |  | <0.001 |
| <25 | 2027(26.41) | 1414(72.83) | 499(23.82) | 109(4.92) | | 5(0.14) |  |
| 25-30 | 2833(35.05) | 506(24.38) | 1158(59.85) | 910(46.43) | | 259(11.98) |  |
| ≥30 | 3105(38.54) | 71(2.80) | 335(16.34) | 971(48.65) | | 1728(87.87) |  |
| TC (mmol/l) | 5.16(0.02) | 5.19(0.03) | 5.23(0.04) | 5.13(0.04) | | 5.07(0.04) | 0.003 |
| HDL-C (mmol/l) | 1.45(0.01) | 1.77(0.01) | 1.49(0.01) | 1.34(0.01) | | 1.19(0.01) | <0.001 |
| LDL-C (mmol/l) | 3.02(0.02) | 2.99(0.03) | 3.15(0.03) | 3.05(0.03) | | 2.91(0.03) | <0.001 |

Continuous variables were expressed as mean (standard error) and categorical variables were expressed as frequencies (percentages).

TyG-WC: triglyceride glucose-waist circumference; PIR: poverty income ratio; HEI: Healthy Eating Index; PA : physical activity; MET: metabolic equivalents; FI: frailty index; BMI: body mass index; TC: total cholesterol; HDL-C: high-density lipoprotein cholesterol; LDL-C: low-density lipoprotein cholesterol.

Supplementary Table 3. Comparison of weighted baseline characteristics of participants according to quartiles of TyG-WHtR

| Variables | Total  N=7965 | TyG-WHtR index | | | | | *P*-value |
| --- | --- | --- | --- | --- | --- | --- | --- |
|  |  | Q1(≤4.73)  N=1993 | Q2(4.73-5.32) N=1990 | | Q3(5.32-6.00) N=1991 | Q4(≥6.00)  N=1991 |  |
| General Characteristics | | | | | | | |
| Gender (%) |  |  |  |  | |  | <0.001 |
| Female | 4075(53.97) | 975(54.99) | 870(48.25) | 1043(53.11) | | 1187(59.49) |  |
| Male | 3890(46.03) | 1018(45.01) | 1120(51.75) | 948(46.89) | | 804(40.51) |  |
| Age (years) | 63.09(0.16) | 61.96(0.30) | 63.32(0.26) | 63.99(0.32) | | 63.31(0.27) | <0.001 |
| Race (%) |  |  |  |  | |  | <0.001 |
| Non-Hispanic Black | 1511(9.30) | 458(10.01) | 391(10.00) | 341(8.82) | | 321(8.24) |  |
| Non-Hispanic White | 3926(76.10) | 1030(77.39) | 964(74.89) | 936(75.25) | | 996(76.68) |  |
| American Mexican | 1124(4.70) | 131(2.09) | 249(4.20) | 364(6.32) | | 380(6.70) |  |
| Others | 1404(9.89) | 374(10.51) | 386(10.91) | 350(9.61) | | 294(8.39) |  |
| Education level (%) |  |  |  |  | |  | <0.001 |
| Below high school | 2213(16.61) | 402(11.69) | 492(15.37) | 632(19.73) | | 687(20.57) |  |
| High school graduate | 1905(25.52) | 436(21.87) | 514(26.41) | 473(27.05) | | 482(27.36) |  |
| College or above | 3847(57.88) | 1155(66.44) | 984(58.22) | 886(53.22) | | 822(52.07) |  |
| PIR (%) |  |  |  |  | |  | <0.001 |
| <1.3 | 1926(14.86) | 390(11.57) | 434(13.92) | 491(14.47) | | 611(20.07) |  |
| 1.3-3.5 | 2936(34.18) | 669(28.56) | 741(33.49) | 771(37.47) | | 755(38.23) |  |
| ≥3.5 | 2410(43.92) | 762(52.39) | 647(45.76) | 542(40.54) | | 459(35.44) |  |
| Unknown | 693(7.04) | 172(7.48) | 168(6.83) | 187(7.52) | | 166(6.25) |  |
| Nutrition & life style | | | | | | | |
| HEI | 55.81(0.26) | 58.21(0.47) | 56.48(0.44) | 54.60(0.42) | | 53.47(0.34) | <0.001 |
| Energy intake (kcal/day) | 1942.03(10.71) | 1991.49(19.30) | 1942.63(20.11) | 1917.43(20.47) | | 1907.56(24.96) | 0.011 |
| Drinking (%) |  |  |  |  | |  | <0.001 |
| Never | 1128(11.41) | 271(10.47) | 250(10.24) | 293(12.50) | | 314(12.64) |  |
| Former | 1752(18.07) | 354(14.08) | 409(16.89) | 469(19.03) | | 520(23.04) |  |
| Mild | 2899(41.90) | 847(47.03) | 794(44.80) | 668(39.50) | | 590(35.27) |  |
| Moderate | 839(12.67) | 222(13.88) | 212(12.55) | 211(11.79) | | 194(12.25) |  |
| Heavy | 796(9.92) | 172(9.17) | 194(10.35) | 207(10.76) | | 223(9.56) |  |
| Unknown | 551(6.02) | 127(5.36) | 131(5.17) | 143(6.43) | | 150(7.25) |  |
| Smoking (%) |  |  |  |  | |  | <0.001 |
| Never | 3902(49.02) | 984(49.58) | 993(51.41) | 994(48.37) | | 931(46.56) |  |
| Former | 2808(35.51) | 622(31.46) | 705(34.58) | 714(38.38) | | 767(38.37) |  |
| Now | 1255(15.47) | 387(18.96) | 292(14.01) | 283(13.25) | | 293(15.07) |  |
| PA (MET-mins/week) |  |  |  |  | |  | <0.001 |
| ≤700 | 1818(23.86) | 483(23.82) | 441(22.37) | 447(25.08) | | 447(24.22) |  |
| 700-2400 | 1851(25.29) | 531(26.89) | 483(27.97) | 454(24.33) | | 383(21.59) |  |
| >2400 | 1783(24.60) | 535(31.51) | 473(25.01) | 426(23.19) | | 349(17.48) |  |
| Unknown | 2513(26.25) | 444(17.78) | 593(24.64) | 664(27.41) | | 812(36.71) |  |
| Medical condition | | | | | | | |
| Lipid-lowering agents (%) |  |  |  |  | |  | <0.001 |
| No | 5036(63.31) | 1457(74.01) | 1272(63.49) | 1221(60.84) | | 1086(53.02) |  |
| Yes | 2929(36.69) | 536(25.99) | 718(36.51) | 770(39.16) | | 905(46.98) |  |
| Hypoglycemic agents (%) |  |  |  |  | |  | <0.001 |
| No | 6452(84.68) | 1873(96.05) | 1733(89.97) | 1585(84.18) | | 1261(66.40) |  |
| Yes | 1513(15.32) | 120(3.95) | 257(10.03) | 406(15.82) | | 730(33.60) |  |
| Frailty (%) |  |  |  |  | |  | <0.001 |
| No | 5559(74.41) | 1604(84.27) | 1535(79.00) | 1382(75.17) | | 1038(57.35) |  |
| Yes | 2406(25.59) | 389(15.73) | 455(21.00) | 609(24.83) | | 953(42.65) |  |
| FI | 0.16(0.00) | 0.13(0.00) | 0.15(0.00) | 0.16(0.00) | | 0.21(0.00) | <0.001 |
| Physical Exam & Laboratory data | | | | | | | |
| BMI (kg/m^2^) |  |  |  |  | |  | <0.001 |
| <25 | 2027(26.41) | 1419(70.50) | 495(23.12) | 104(3.88) | | 9(0.22) |  |
| 25-30 | 2833(35.05) | 528(28.01) | 1176(60.89) | 912(43.11) | | 217(8.84) |  |
| ≥30 | 3105(38.54) | 46(1.49) | 319(15.99) | 975(53.01) | | 1765(90.94) |  |
| TC (mmol/l) | 5.16(0.02) | 5.10(0.03) | 5.22(0.04) | 5.19(0.03) | | 5.13(0.04) | 0.055 |
| HDL-C (mmol/l) | 1.45(0.01) | 1.71(0.01) | 1.48(0.01) | 1.35(0.01) | | 1.22(0.01) | <0.001 |
| LDL-C (mmol/l) | 3.02(0.02) | 2.97(0.03) | 3.12(0.03) | 3.07(0.03) | | 2.94(0.03) | <0.001 |

Continuous variables were expressed as mean (standard error) and categorical variables were expressed as frequencies (percentages).

TyG-WHtR: triglyceride glucose-waist to height ratio; PIR: poverty income ratio; HEI: Healthy Eating Index; PA : physical activity; MET: metabolic equivalents; FI: frailty index; BMI: body mass index; TC: total cholesterol; HDL-C: high-density lipoprotein cholesterol; LDL-C: low-density lipoprotein cholesterol.

Supplementary Table 4. Comparison of weighted baseline characteristics of participants according to quartiles of TyG-BMI

| Variables | Total  N=7965 | TyG-BMI index | | | | | *P*-value |
| --- | --- | --- | --- | --- | --- | --- | --- |
|  |  | Q1(≤213.94)  N=1991 | Q2(213.94-248.53) N=1992 | | Q3(248.53-291.70) N=1990 | Q4(≥291.70)  N=1992 |  |
| General Characteristics | | | | | | | |
| Gender (%) |  |  |  |  | |  | <0.001 |
| Female | 4075(53.97) | 1040(59.21) | 899(48.39) | 984(51.67) | | 1152(56.06) |  |
| Male | 3890(46.03) | 951(40.79) | 1093(51.61) | 1006(48.33) | | 840(43.94) |  |
| Age (years) | 63.09(0.16) | 63.50(0.35) | 63.65(0.27) | 63.27(0.26) | | 61.94(0.27) | <0.001 |
| Race (%) |  |  |  |  | |  | <0.001 |
| Non-Hispanic Black | 1511(9.30) | 352(7.81) | 367(9.35) | 380(9.79) | | 412(10.39) |  |
| Non-Hispanic White | 3926(76.10) | 1067(77.88) | 956(74.95) | 967(76.32) | | 936(75.12) |  |
| American Mexican | 1124(4.70) | 141(2.23) | 292(4.79) | 334(5.79) | | 357(6.22) |  |
| Others | 1404(9.89) | 431(12.07) | 377(10.91) | 309(8.10) | | 287(8.26) |  |
| Education level (%) |  |  |  |  | |  | <0.001 |
| Below high school | 2213(16.61) | 462(13.98) | 542(16.05) | 595(18.48) | | 614(18.16) |  |
| High school graduate | 1905(25.52) | 458(22.64) | 466(24.91) | 509(28.40) | | 472(26.43) |  |
| College or above | 3847(57.88) | 1071(63.38) | 984(59.04) | 886(53.11) | | 906(55.42) |  |
| PIR (%) |  |  |  |  | |  | 0.002 |
| <1.3 | 1926(14.86) | 441(13.33) | 451(13.93) | 476(14.54) | | 558(17.71) |  |
| 1.3-3.5 | 2936(34.18) | 687(30.55) | 770(34.75) | 745(35.80) | | 734(35.95) |  |
| ≥3.5 | 2410(43.92) | 690(48.79) | 603(43.69) | 592(42.86) | | 525(39.97) |  |
| Unknown | 693(7.04) | 173(7.33) | 168(7.63) | 177(6.79) | | 175(6.37) |  |
| Nutrition & life style | | | | | | | |
| HEI | 55.81(0.26) | 58.32(0.53) | 56.71(0.39) | 54.82(0.41) | | 53.17(0.37) | <0.001 |
| Energy intake (kcal/day) | 1942.03(10.71) | 1933.35(17.77) | 1923.41(24.40) | 1937.64(21.86) | | 1973.78(22.08) | 0.436 |
| Drinking (%) |  |  |  |  | |  | 0.022 |
| Never | 1128(11.41) | 299(11.75) | 271(10.70) | 266(11.59) | | 292(11.57) |  |
| Former | 1752(18.07) | 376(15.22) | 405(16.53) | 469(19.04) | | 502(21.72) |  |
| Mild | 2899(41.90) | 798(44.04) | 767(45.24) | 716(40.43) | | 618(37.75) |  |
| Moderate | 839(12.67) | 213(13.98) | 208(11.44) | 213(12.95) | | 205(12.21) |  |
| Heavy | 796(9.92) | 167(9.41) | 199(9.97) | 199(10.34) | | 231(10.04) |  |
| Unknown | 551(6.02) | 138(5.61) | 142(6.11) | 127(5.65) | | 144(6.71) |  |
| Smoking (%) |  |  |  |  | |  | <0.001 |
| Never | 3902(49.02) | 978(48.51) | 998(51.29) | 967(47.12) | | 959(49.16) |  |
| Former | 2808(35.51) | 614(30.98) | 693(35.38) | 748(38.88) | | 753(37.24) |  |
| Now | 1255(15.47) | 399(20.50) | 301(13.33) | 275(13.99) | | 280(13.61) |  |
| PA (MET-mins/week) |  |  |  |  | |  | <0.001 |
| ≤700 | 1818(23.86) | 475(23.67) | 438(22.93) | 439(23.34) | | 466(25.47) |  |
| 700-2400 | 1851(25.29) | 517(27.70) | 472(26.26) | 454(24.58) | | 408(22.43) |  |
| >2400 | 1783(24.60) | 465(27.63) | 502(27.52) | 442(24.10) | | 374(18.99) |  |
| Unknown | 2513(26.25) | 534(21.01) | 580(23.30) | 655(27.97) | | 744(33.12) |  |
| Medical condition | | | | | | | |
| Lipid-lowering agents (%) |  |  |  |  | |  | <0.001 |
| No | 5036(63.31) | 1406(71.11) | 1285(66.01) | 1208(59.95) | | 1137(55.54) |  |
| Yes | 2929(36.69) | 585(28.89) | 707(33.99) | 782(40.05) | | 855(44.46) |  |
| Hypoglycemic agents (%) |  |  |  |  | |  | <0.001 |
| No | 6452(84.68) | 1840(94.87) | 1711(88.80) | 1581(84.61) | | 1320(69.81) |  |
| Yes | 1513(15.32) | 151(5.13) | 281(11.20) | 409(15.39) | | 672(30.19) |  |
| Frailty (%) |  |  |  |  | |  | <0.001 |
| No | 5559(74.41) | 1571(82.36) | 1514(79.90) | 1381(73.26) | | 1093(61.60) |  |
| Yes | 2406(25.59) | 420(17.64) | 478(20.10) | 609(26.74) | | 899(38.40) |  |
| FI | 0.16(0.00) | 0.14(0.00) | 0.15(0.00) | 0.17(0.00) | | 0.20(0.00) | <0.001 |
| Physical Exam & Laboratory data | | | | | | | |
| BMI (kg/m^2^) |  |  |  |  | |  | <0.001 |
| <25 | 2027(26.41) | 1699(84.95) | 318(15.00) | 10(0.25) | | 0(0.00) |  |
| 25-30 | 2833(35.05) | 292(15.05) | 1589(81.36) | 899(44.52) | | 53(1.94) |  |
| ≥30 | 3105(38.54) | 0(0.00) | 85(3.64) | 1081(55.23) | | 1939(98.06) |  |
| TC (mmol/l) | 5.16(0.02) | 5.13(0.03) | 5.21(0.03) | 5.21(0.03) | | 5.08(0.03) | 0.004 |
| HDL-C (mmol/l) | 1.45(0.01) | 1.73(0.02) | 1.47(0.01) | 1.34(0.01) | | 1.24(0.01) | <0.001 |
| LDL-C (mmol/l) | 3.02(0.02) | 2.95(0.03) | 3.13(0.03) | 3.10(0.03) | | 2.92(0.03) | <0.001 |

Continuous variables were expressed as mean (standard error) and categorical variables were expressed as frequencies (percentages).

TyG-BMI: triglyceride glucose-body mass index; PIR: poverty income ratio; HEI: Healthy Eating Index; PA : physical activity; MET: metabolic equivalents; FI: frailty index; BMI: body mass index; TC: total cholesterol; HDL-C: high-density lipoprotein cholesterol; LDL-C: low-density lipoprotein cholesterol.
